# The Russian registry of primary hyperparathyroidism, latest update

**DOI:** 10.3389/fendo.2023.1203437

**Published:** 2023-07-03

**Authors:** Natalia G. Mokrysheva, Anna K. Eremkina, Alina R. Elfimova, Elena V. Kovaleva, Anastasiia P. Miliutina, Ekaterina E. Bibik, Anna M. Gorbacheva, Ekaterina A. Dobreva, Irina S. Maganeva, Julia A. Krupinova, Rustam H. Salimkhanov, Lizaveta A. Aboishava, Elena V. Karaseva, Galina A. Melnichenko, Ivan I. Dedov

**Affiliations:** ^1^ Directorate, Endocrinology Research Centre, Moscow, Russia; ^2^ Department of Parathyroid Glands Pathology, Endocrinology Research Centre, Moscow, Russia

**Keywords:** prevalence, registry, hypercalcemia, parathyroid hormone, primary hyperparathyroidism

## Abstract

**Introduction:**

Until recently no major epidemiological research of primary hyperparathyroidism (PHPT) has been conducted in the Russian Federation, this led to the creation of the Russian online registry. The objective of this study is to estimate the clinical and biochemical profile, classical and non-classical complications, surgical intervention and medical therapy of the patients with different forms of PHPT in the Russian Federation.

**Materials and methods:**

The cross-sectional, observational, continuous study was conducted at the Endocrinology Research Centre (Moscow). The present study explored retrospective data from 6003 patients submitted to the Registry between 12.12.2016 and 25.10.2022 from 81 regions of the Russian Federation (http://pgpt.clin-reg.ru/).

**Results:**

The median age was 59 [60; 66] years with a female:male ratio of 11.7:1. Symptomatic PHPT was observed in 74.3% while asymptomatic form - only in 25.7% of cases. Bone pathology was the predominant clinical manifestation in 62.5% of cases (n=2293), mostly in combination with visceral complications 45.7% (n=1676). The majority of patients (63.3%) had combined visceral disorders including kidney damage in 51.8% and gastroduodenal erosions/ulcers in 32.3% of patients. Symptomatic patients were older (60 [53; 67] vs. 54 [45; 62] years, p<0.001) and had more severe biochemical alterations of calcium-phosphorus metabolism. Cardiovascular disease (СVD) was recorded in 48% of patients, among them the most frequent was arterial hypertension (up to 93.9%). A genetic test was conducted in 183 cases (suspicious for hereditary PHPT) revealing the mutations in *MEN1*, *CDC73*, *RET* genes in 107, 6 and 2 cases, respectively. Surgery was performed in 53.4% of patients with remission achievement in 87%, the relapse/persistence were recorded in 13% of cases. Histological examination revealed carcinoma in 4%, atypical adenoma in 2%, adenoma in 84% and hyperplasia in 11% of cases. Drug therapy was prescribed in 54.0% of cases, most often cholecalciferol.

**Conclusion:**

The detection rate of PHPT has increased in the Russian Federation in recent years. This increase is associated with the start of online registration. However, the majority of patients remain symptomatic with significant alterations of phosphorus-calcium metabolism that indicates delayed diagnosis and requires further modifications of medical care.

## Introduction

Primary hyperparathyroidism (PHPT) is a relatively common endocrine disease caused by excessive secretion of parathyroid hormone (PTH) by pathologically altered parathyroid glands. PHPT is usually caused by a solitary parathyroid adenoma (80% of cases), whereas hyperplasia accounts for 10–15%, multiple adenomas for 5% and parathyroid cancer for 1% of cases (1). In countries where routine evaluation of serum calcium levels became widespread the incidence of PHPT has increased during the past decades. However, the prevalence remains variable by regions and race, and ranges in 0.4-82 cases per 100,000 (2–4).Usually, PHPT predominates among postmenopausal women, with a female/male ratio of 3 to 4:1. Most often, PHPT is a sporadic disease, with no family history and no evidence for other endocrine gland involvement. Hereditary forms amount to about 10% of the PHPT population, and can be limited to the parathyroid glands or be a part of multiple endocrine syndromes (1).

Chronic hypercalcemia and the catabolic effect of excessive PTH are considered as the main pathogenic mechanisms of the disease. Classical PHPT characterized by symptomatic course with severe skeletal, renal and gastrointestinal complications is uncommon in North America and Western Europe. In these regions the most typical feature of PHPT is the asymptomatic hypercalcemia, which usually is an incidental finding on routine biochemical screening and usually is within 0,25 mmol/l (1 mg/dl) above the upper limit of normal range (5). However, in developing countries, PHPT is more often diagnosed only when symptoms are present (6, 7).

Much less is known about the non-classical PHPT complications. For a long time, PTH has been considered as a hormone that regulates only calcium homeostasis in bone and kidney. But along with the “classical” ones, there are other “nonclassical” target cells, including erythrocytes and lymphocytes, hepatocytes, smooth muscle cells, adipocytes and cardiomyocytes. PHPT may be associated with increased cardiovascular morbidity and mortality, even in mild disease (8). Moreover, the cognitive and psychological symptoms as well as cardiovascular diseases are the part of the “asymptomatic” form of PHPT that is the predominant today. Experts concluded that patients with asymptomatic PHPT have many cognitive complaints, cardiovascular and metabolic disorders, but data regarding their precise nature and reversibility are inconsistent (5). Normocalcemic PHPT was described in the first decade of the 21st century but still is a big challenge. It must be diagnosed by exclusion, laboratory testing over time is necessary to distinguish normocalcemic PHPT from secondary hyperparathyroidism (SHPT). In some cases, differential diagnostic tests should be used to verify the diagnosis (9). Normocalcemic PHPT can be asymptomatic or symptomatic, thus different treatment strategies can be applied. However, evidence on the effect of parathyroidectomy (PTx) or any other management of normocalcemic PHPT is scarce, and there are no clear data on the natural history of normocalcemic PHPT (10). Until recently no major epidemiological research has been conducted in the Russian Federation. This led to creation of the Russian online registry of PHPT with accessibility from various regions of the country.

The objective of this study is to estimate the clinical and biochemical profile, classical and non-classical complications, surgical intervention and medical therapy of the patients with different forms of PHPT in the Russian Federation.

## Materials and methods

### Study design

The cross-sectional, observational, continuous study was conducted at the Endocrinology Research Centre (Moscow). The technical development of the Registry and start of the registration with online input of data were carried out in 2016. Nowadays the Registry is available online at http://pgpt.clin-reg.ru/. The present study explored retrospective data from 6003 patients submitted to the Registry between 12.12.2016 and 25.10.2022 from 81 regions of the Russian Federation. The analysis included the first data entry into the Registry (the «first visit») and the last one (the “last visit”) with dynamic data when available. More than half of the records are patients from Moscow and the Moscow region - 3400 persons (56.6%). The Tyumen region was in second place in terms of the number of patients with PHPT (n=475, 7.9%).

The Registry was created in accordance with the Russian clinical guidelines. This database included subjects with PHPT of all ages and of any etiology. The diagnosis of PHPT was confirmed on the results of laboratory tests: an elevated serum PTH level in combination with serum albumin adjusted calcium >2.55 mmol/L (at least twice in consecutive measurements) or with normocalcemia after SHPT was ruled out. The criteria for an asymptomatic form of PHPT without target organ involvement [similar to the Fifth International Workshop on PHPT (11)] were a mildly increased blood calcium (within 0.25 mmol|l =1 mg/dl above the upper limit of the reference range), hypercalciuria less than 10 mmol/day (400 mg/day); no history and/or no radiologically verified low-traumatic fractures; no significant decrease in bone mineral density (BMD, not less than -2.0 SD in Z-score or -2.5 SD in T-score), the absence of nephrolithiasis/nephrocalcinosis, glomerular filtration rate (GFR) more than 60 ml/min/1.73 m^2^. Patients with clinically significant hypercalcemia and hypercalciuria, confirmed skeletal and/or renal complications according to the Russian Guidelines were considered as symptomatic. All clinical data were collected anonymously, using an online medical record on the registry platform. Informed consent was collected in accordance with General Authorization to Process Personal Data for Scientific Research Purposes.

All patients were registered in the specialized endocrinological centers. In each included center, the responsible endocrinologists submitted the data online with personal access code to self-entry of data. All clinical data in the Registry has been obtained from the baseline evaluation reported in clinical records of each center (including the Endocrinology Research Centre) and with dynamic follow-ups when available. Then, all data were collected and analyzed by the Endocrinology Research Centre as a coordinating institution.

The detailed clinical history of each patient at the time of presentation was recorded. The following data was analyzed: demographic parameters (sex, age, geographic region); age of onset of the disease; biochemical evaluation (albumin-adjusted (calculated automatically) and ionized serum calcium, serum phosphorus, serum iPTH, serum creatinine (estimated GFR was calculated automatically by Chronic Kidney Disease Epidemiology Collaboration (CKD-EPI) formula); alkaline phosphatase (ALP); 24h urinary calcium; genetic tests in case of hereditary PHPT (if available); the classical and nonclassical (cardiovascular) complications; the preoperative localization of parathyroid lesion(s) with neck ultrasonography and 99mTc-sestamibi scintigraphy and/or contrast computed tomography (CT), surgery with histopathological confirmation of typical adenoma/atypical adenoma/hyperplasia/carcinoma; medical therapy (if available); treatment outcomes. For all laboratory parameters, the reference interval (RI) of the local laboratory was used with the obligatory indication of the lower and upper limits. The presence or absence of PHPT complications was based on the results of laboratory and instrumental examination. Renal manifestations in PHPT patients were confirmed by the history of renal colic, renal stone or/and positive radiological parameters of nephrolithiasis/nephrocalcinosis identified either by ultrasonography or by CT. The diagnosis of osteoporosis was based on the results of the radiography and dual X-ray absorptiometry. BMD was measured at the lumbar spine (LS), femoral neck (FN), total hip (TH) and radius 33% (R33%) and assessed by Т- and Z-score regarding age and reproductive function. The presence of concomitant cardiovascular disorders was based on cardiologist consultation (when available).The decision of labeling the «Suspicion of the hereditary PHPT» field was carried out by the responsible endocrinologists. According to the Russian guidelines for PHPT, the Registry allows to evaluate the following factors: multiple lesions of the parathyroid glands, recurrence of the disease, age <40 years, the presence of other significant endocrine pathology (pituitary adenoma, neuroendocrine tumor (NET), adrenal adenoma, medullary thyroid carcinoma, pheochromocytoma), family history. The genetic study was performed in the Endocrinology Research Centre. Screening for *MEN1*, *RET* and *CDC73* gene mutations was done by Sanger sequencing or by next-generation sequencing using the panel of all these genes. Sequencing of the gene panel was carried out on the Illumina NextSeq 550 platform (Illumina, USA), in the case of Sanger sequencing - on the AB3500 genetic analyzer (Thermo Fisher Scientific, USA). We applied paired-end (PE) reads (minimum coverage depth × 100) strategy. Genomic coordinates refer to the latest version GRCh38.There were no specific factors that could affect the external generalization of the research findings.

Sample size calculation principles: sample size calculation was not required.

### Statistical analysis

Statistical analysis was performed using Statistica 13.0 (StatSoft, USA) software package. Descriptive statistics of quantitative characteristics are presented by medians and lower, upper quartiles (Median [Q1; Q3], descriptive statistics of qualitative characteristics - in absolute and relative frequencies [n (%)].The Mann – Whitney test (U-test) was applied to compare two independent groups in terms of quantitative characteristics due to the fact that it is suitable for any type of distribution of quantitative characteristics. The Fisher exact test was applied to compare two independent groups in terms of qualitative characteristics because it can be used for any values of absolute and expected frequencies. The critical level of significance when testing statistical hypotheses was taken equal to 0.05. In multiple comparisons, the Bonferroni correction was applied by correcting the critical level of significance.

## Results

**Figure 1:**
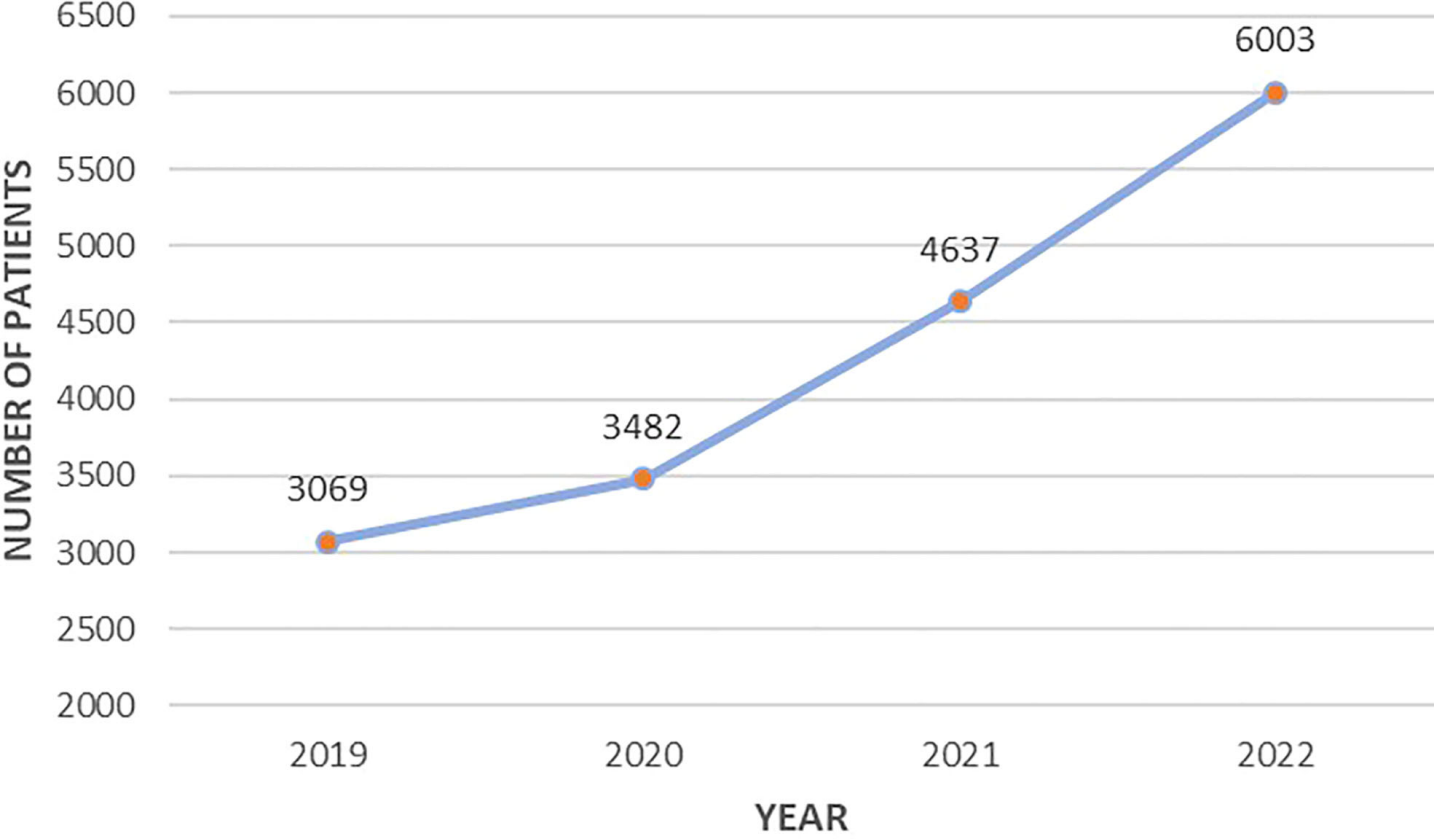
Cumulative number of registered PHPT patients by year from 2019 to 2022.

Since 2019, there has been a positive trend in the Registry data entry, which is associated with the consistent connection of regions to the online platform as well as the increased detection rate of PHPT in the Russian Federation (**Figure 1**).

**Figure 2:**
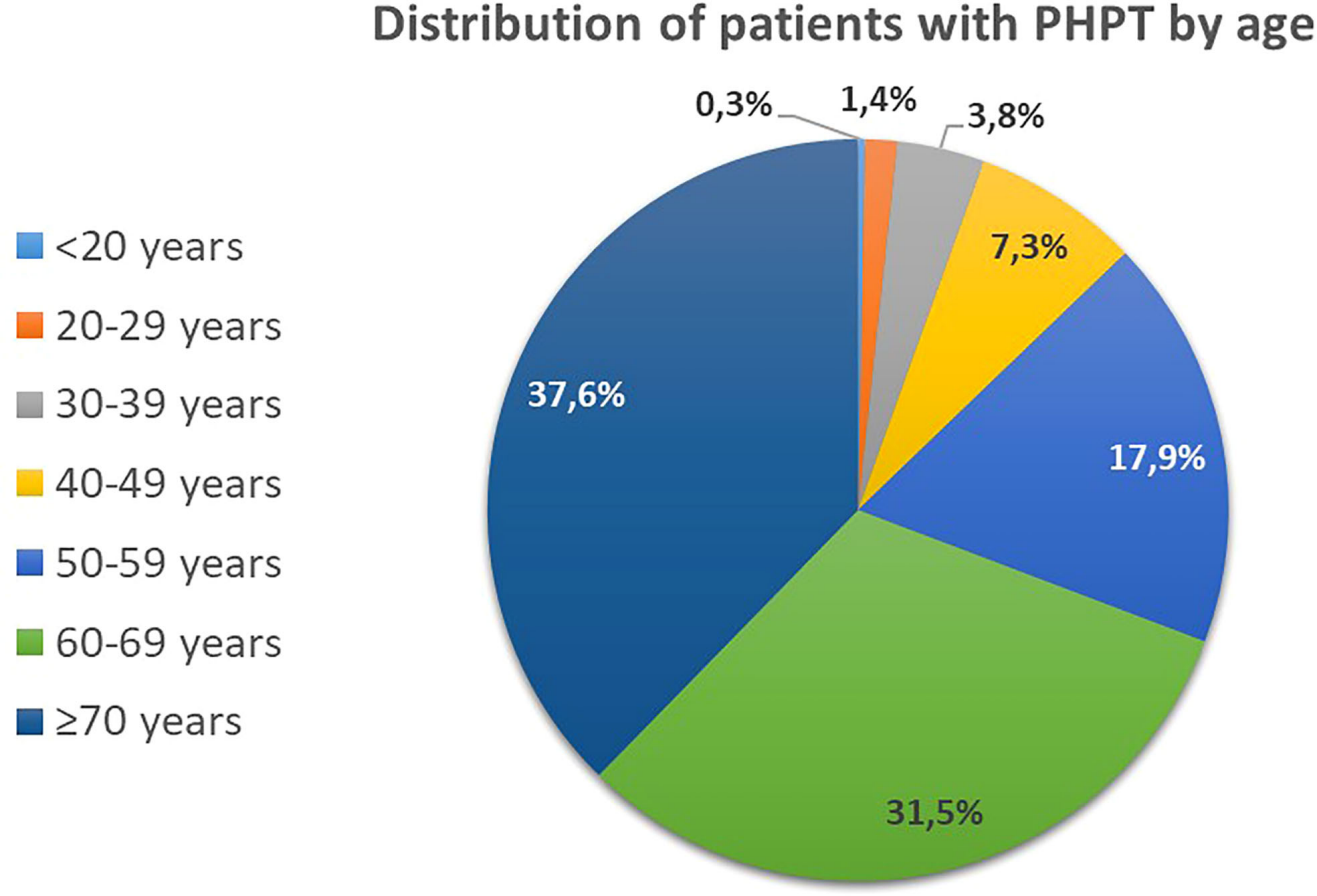
Distribution of patients with PHPT by age.

The median age in the common group was 59 [60; 66] years with a female: male ratio of 11.7:1. The age distribution is shown in **Figure 2**. The age at the time of diagnosis in men was lower than in women - 52 years [37; 64] vs. 59 years [51; 66] (p<0.001, U-test). On the first visit the active phase of PHPT was registered in 82.2% (n=4934) of patients, relapse - in 1.8% (n=90), remission - in 8.1% (n=491); in 8.1% (n=488) of cases the data on disease activity was absent in the record.

Among patients with active disease, symptomatic PHPT was observed in 74.3% while asymptomatic form - only in 25.7% of cases. The predominant clinical manifestation was bone pathology in 62.5% of cases (n=2293), mostly in combination with visceral complications (45.7%, n=1676). The isolated skeletal disorders were noted in 16.8% (n=617). 41.1% (n=944) patients had low-energy fractures. Bone involvement was more frequent in women compared to men (64.0 vs. 45.4%, р<0.001, Fisher exact test). Median values of DXA are presented in **Table 1**.

**Table 1:** T-score and Z-score in patients with PHPT (n=2293).

	Z-score		T-score	
	N	Median [Q1; Q3]	N	Median [Q1; Q3]
Femur Neck	160	-2.1 [-3.1; -1.2]	1287	-2.1 [-2.6; -1.5]
L1-L4	188	-2.5 [-3.1; -1.5]	1502	-2.7 [-3.3; -1.8]
Radius 33%	116	-2.9 [-4.1; -2.2]	875	-3.2 [-4.0; -2.6]
Radius Total	98	-2.6 [-4.2; -1.5]	700	-3.3 [-4.2; -2.6]
Total hip	106	-1.9 [-2.6; -0.8]	822	-1.7 [-2.5; -1.1]

Data are presented by medians and interquartile ranges (Median, IQR [25;75]%). N - Number of people examined. PHPT, primary hyperparathyroidism; PTH, parathyroid hormone.

The majority of patients (63.3%, n=2320) had combined visceral disorders. Herewith visceral complications included structural and/or functional kidney damage in 51.8% (n=1898) and gastroduodenal erosions/ulcers - in 32.3% (n=1147) of patients. Kidney pathology was generally more common in men compared to women (63.1 vs. 50.8%, р<0.001, Fisher exact test). Nephrolithiasis was recorded in 44.8% (n=851) cases, while nephrocalcinosis - in 2.8% (n=54) cases. GFR data was available in 1457 patients. Distribution by CKD study was as follows: C1 - 17.1% (n=249), C2 - 40.5% (n=590), C3a - 27.0% (n=394), C3b - 9.8% (n=143), C4 - 4.4% (n=64), C5 - 0.8% (n=12). Symptomatic and asymptomatic patients differed in age (60 [53; 67] vs. 54 [45; 62] years, p<0.001). Serum iPTH, albumin adjusted and ionized Cа levels, ALP, as well as daily calciuria were higher in symptomatic patients (p<0.001 for all). Severe hypophosphatemia and a more pronounced decrease in GFR were also more typical for the symptomatic PHPT (p<0.001 for both). The data is presented in **Table 2**.

**Table 2:** Clinical features of symptomatic and asymptomatic PHPT (n=4934).

Variable	Symptomatic PHPT (N=3666)		Asymptomatic PHPT (N=1268)		p
	N	Median [Q_1_; Q_3_] or N,%.	N	Median [Q_1_; Q_3_] or N,%.	
Male	3666	295 (8.1%)	1268	100 (7.9%)	0.905^2^
Age of manifestation, years	3666	60 [53; 67]	1268	54 [45; 62]	<0.001^1^
Calcium total, mmol/l	3456	2.77 [2.64; 2.94]	1173	2.69 [2.58; 2.80]	<0.001^1^
Albumin-adjusted calcium, mmol/l	832	2.71 [2.60; 2.88]	221	2.62 [2.52; 2.71]	<0.001^1^
Calcium ionized, mmol/l	2603	1.39 [1.30; 1.50]	908	1.32 [1.22; 1.41]	<0.001^1^
Phosphorus, mmol/l	2568	0.87 [0.75; 1.00]	768	0.90 [0.80; 1.04]	<0.001^1^
PTH, pg/ml	3633	155.0 [101.0; 269.0]	1254	121.9 [91.7; 167.0]	<0.001^1^
ALP, units/l	1902	126.6 [87.0; 236.0]	518	102.0 [75.0; 170.0]	<0.001^1^
Urinary calcium 24 h, mmol/24 h	1617	7.67 [5.05; 10.49]	439	7.28 [5.02; 9.80]	0.176^1^
Creatinine, mcmol/l	2556	74.0 [64.7; 88.0]	711	71.0 [63.0; 80.2]	<0.001^1^
GFR, ml/min/1.73m2	2572	76 [61; 90]	720	85 [71; 98]	<0.001^1^

^1^U-test; ^2^Fisher exact test; Bonferroni correction Р_0 _= 0.05/16 = 0.003. Data are presented by medians and interquartile ranges (Median, IQR [25;75]%). N - Number of people examined. PHPT, primary hyperparathyroidism; PTH, parathyroid hormone; GFR, estimated glomerular filtration rate; ALP, alkaline phosphatase.

According to the Registry, normocalcemic PHPT (nPHPT) was diagnosed in 5.8% cases. The comparative analysis included patients with all available calcium parameters within RR for nPHPT and all available calcium parameters above RR for the hypercalcemic group. Patients with nPHPT had lower serum calcium, daily calciuria and iPTH values, as well as higher phosphorus levels. At the same time, subgroups with normocalcemic and hypercalcemic variants had similar frequencies of bone and visceral complications. The data is presented in **Table 3**.

**Table 3:** Clinical features of normocalcemic and hypercalcemic PHPT (n=1914).

Variable	Normocalcemic PHPT (N=346)		Hypercalcemic PHPT (N=1568)		p
	N	Me [Q_1_; Q_3_] or N,%.	N	Me [Q_1_; Q_3_] or N,%.	
Male	346	27 (7.8%)	1568	156 (9.9%)	0.266^2^
Age of manifestation, years	346	57 [48; 66]	1568	56 [48; 63]	0.073^1^
Calcium total, mmol/l	329	2.44 [2.32; 2.51]	1466	2.82 [2.70; 3.01]	<0.001^1^
Albumin-adjusted calcium, mmol/l	40	2.39 [2.20; 2.45]	209	2.77 [2.65; 2.91]	<0.001^1^
Calcium ionized, mmol/l	302	1.17 [1.11; 1.23]	1160	1.40 [1.30; 1.52]	<0.001^1^
Phosphorus, mmol/l	225	0.98 [0.87; 1.10]	1007	0.82 [0.70; 0.96]	<0.001^1^
PTH, pg/ml	346	127.85 [97.5; 182]	1568	182.35 [126; 334]	<0.001^1^
Urinary calcium 24 h, mmol/24 h	123	6.2 [4.1; 9.3]	554	8.6 [5.9; 11.3]	<0.001^1^
Symptomatic PHPT	346	346 (100%)	1568	1568 (100%)	1.000^2^
Isolated bone complication	346	147 (42.5%)	1568	780 (49.7%)	0.015^2^
Isolated kidney complication	346	118 (34.1%)	1568	574 (36.6%)	0.388^2^
Combination of visceral and bone involvement	346	60 (17.3%)	1568	327 (20.9%)	0.160^2^
Cardiovascular diseases during life	346	151 (43.6%)	1568	710 (45.3%)	0.592^2^

^1^U-test; ^2^Fisher exact test; Bonferroni correction Р_0 _= 0.05/13 = 0.004. Data are presented by medians and interquartile ranges (Median, IQR [25;75]%). N - Number of people examined. PHPT, primary hyperparathyroidism; PTH, parathyroid hormone.

Cardiovascular disease (СVD) was recorded in 2366 (48.0%) of patients. Body mass index was significantly higher in the group with CVD - 29.34 kg/m2 [25.80; 33.73] vs. 26.30 kg/m2 [23.28; 30.10], p<0.001 (U-test). Among patients with different CVD the most common CVD was arterial hypertension (AH) (93.9%, n=2221), while left ventricular hypertrophy was present only in 5.6% (n=133) of patients. A history of coronary artery disease (CAD) was detected in 19.6% (n=464), calcification of the structures of the heart and/or blood vessels - in 2.7% (n=63) of patients. The asymptomatic PHPT was characterized by the lower frequency of CVD (Bonferroni correction p0 = 0.003): for all CVD 37.0% (n=469) vs. 51.8% (n=1897), p<0.001 (Fisher exact test), for AH 35.0% (n=444) vs. 48.5% (n=1777), p<0,001 (Fisher exact test) and for ischemic heart disease 4,0% (n=51) vs. 21.8% (n=413), p<0.001. In asymptomatic PHPT there was a tendency to lower frequency of left ventricular hypertrophy - 1.9% (n=24) vs. 3.0% (n=109), p=0.044 (Fisher exact test) and calcification of the structures of the heart and/or blood vessels - 0.6% (n=7) vs. 1.5% (n=56), p=0.006 (Fisher exact test). Patients with AH were significantly older (62 vs. 55 years, р<0.001, U-test), had higher serum PTH levels (148.8 vs. 137.8 pg/ml, p<0.001, U-test), and lower GFR (67 vs. 74 ml/min/1.73m2, p<0.001, U-test) compared to normotonic patients. Patients with CAD were also older than those without (67 vs. 58 years, р<0.001, U-test) and had higher ionized calcium levels (1.40 vs. 1.37 mmol/l, p =0.006), and lower GFR (66 vs. 80 ml/min/1.73m^2^, p<0.001, U-test).

**Figure 3:**
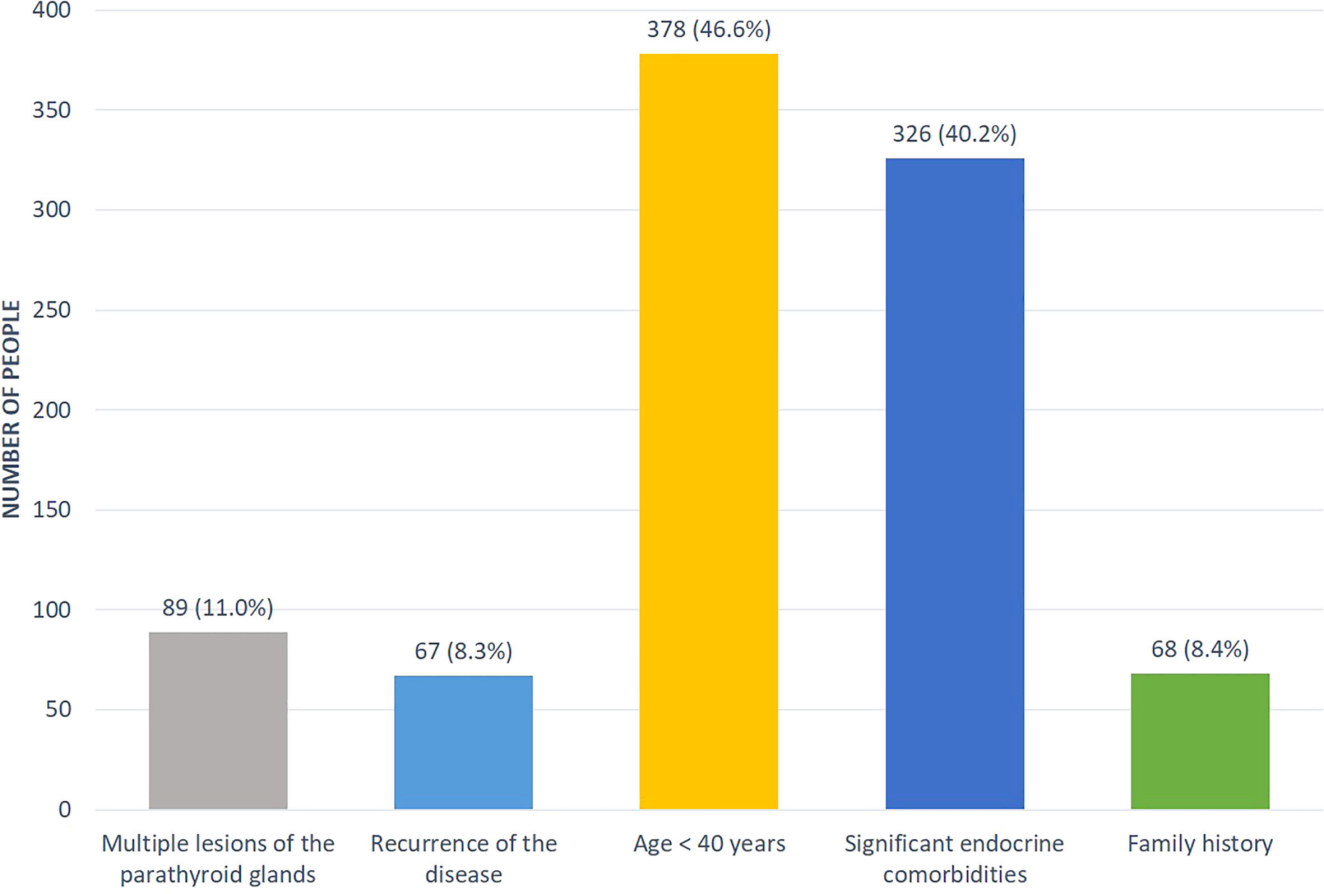
The distribution of suspicious factors for hereditary PHPT.

Some patients (n=811, 14%) had a suspicion for hereditary PHPT (predominantly in relation to MEN1 syndrome). The distribution by reasons was presented in the **Figure 3**. A genetic analysis was conducted in 183 cases (23%) revealing the mutations in *MEN1, CDC73, RET* genes in 107, 6 and 2 cases, respectively. Among patients with *MEN1* mutations, family anamnesis was positive in 26 (24.3%) cases. As expected, the PHPT onset in the MEN-1 group was earlier compared to sporadic disease (33 [26; 48] vs. 59 [52; 66] years, p<0.001), the average duration of disease before diagnosis did not differ. The presence of two or more MEN-1-associated neoplasms was determined in 87.9% (n=94) cases. The most frequent components were pituitary adenoma 59.8% (n=64), pancreatic tumors 53.3% (n=57) and adrenal adenoma 42.1% (n=45), while isolated PHPT was diagnosed in 12.2% (n=13). We did not find any differences in the iPTH, phosphorus and creatinine levels, but there were tendencies to more severe hypercalcemia and a less frequent combination of visceral and bone involvement in MEN-1 group (**Table 4**). 67.1% of MEN-1 patients had symptomatic PHPT, more often with bone manifestation (35.1%). Multiple parathyroid involvement at the time of primary diagnosis was found in 27.1% (n=29) patients with MEN-1. Primary surgical treatment was performed in 77.6% (n=83) of these patients, 28.9% (n=24) relapsed.

**Table 4:** Clinical features of sporadic and MEN1-associated PHPT (n=4934).

Variable	Sporadic PHPT (N=4849)		PHPT in MEN-1 (N=85, only patients in active phase were included)		p
	N	Me [Q_1_; Q_3_] or N, %	N	Me [Q_1_; Q_3_] or N, %	
Male	4849	372 (7.7%)	85	23 (27.1%)	<0.001^2^
Age of manifestation, years	4849	59 [51; 66]	85	32 [25; 47]	<0.001^1^
Calcium total, mmol/l	4557	2.74 [2.62; 2.90]	81	2.81 [2.67; 2.97]	0.027^1^
Albumin-adjusted calcium, mmol/l	1044	2.69 [2,58; 2,85]	9	2.60 [2.49; 2.68]	0.167^1^
Calcium ionized, mmol/l	3439	1.37 [1.28; 1.48]	72	1.32 [1.24; 1.44]	0.030^1^
Phosphorus, mmol/l	3281	0.88 [0.76; 1.00]	55	0.90 [0.76; 1.06]	0.459^1^
PTH, pg/ml	4802	143.9 [97.8; 232.0]	85	147.0 [100.0; 231.6]	0,747^1^
Symptomatic PHPT	4849	3609 (74.4%)	85	57 (67.1%)	0,133^2^
Isolated bone complications	3609	1203 (33.3%)	57	20 (35.1%)	0,778^2^
Isolated kidney complications	3609	812 (22.5%)	57	16 (28.1%)	0,338^2^
Combination of visceral and bone involvement	3609	1062 (29.4%)	57	8 (14.0%)	0,012^2^
Multiple enlarged parathyroid glands	4849	351 (7.2%)	85	26 (30.6%)	<0,001^2^

^1^ U-test; ^2^ Fisher exact test; Bonferroni correction Р0 = 0,05/12 = 0,004. Data are presented by medians and interquartile ranges (Median, IQR [25;75]%). N - Number of people examined. PHPT, primary hyperparathyroidism; MEN-1, Multiple Endocrine Neoplasia Type 1; PTH, parathyroid hormone.

The data about the imaging methods of the parathyroid glands was available for 4934 patients (**Table 5**). Surgical treatment was performed in 3207 patients (53.4%) with remission achievement in 87%, the relapse or persistence after surgery were recorded in 13%. Histological examination was available for 2666 (83%) patients. Parathyroid carcinoma (PC) was verified in 4% of cases (n=101), atypical adenoma in 2% (n=53), adenoma in 84% (n=2227), hyperplasia in 11% (n=285). Сarcinoma was more common in men. Compared to the adenoma group, patients with PC had higher levels of PTH, ionized and total calcium, ALP, creatinine, and the lower GFR. The frequency of kidney pathology was higher in the carcinoma group versus the adenoma group (56.6% vs. 34.9%, **Table 6**).

**Table 5:** The imaging methods of the parathyroid glands according to the Registry.

Imaging method of the parathyroid glands	Number of people examined (N, %)
Ultrasound	1512 (30.6%)
Scintigraphy	408 (8.3%)
CT	102 (2.1%)
Ultrasound & Scintigraphy	1551 (31.4%)
Ultrasound & CT	360 (7.3%)
Scintigraphy & CT	90 (1.8%)
Ultrasound & Scintigraphy & CT	372 (7.5%)

CT, Computed Tomography.

**Table 6:** Clinical features of parathyroid adenoma and parathyroid carcinoma (n = 1850).

Variable	Parathyroid adenoma (N=1767)		Parathyroid carcinoma (N=83)		p
	N	Median [Q_1_; Q_3_] or N,%.	N	Median [Q_1_; Q_3_] or N,%.	
Male	1767	159 (9.0%)	83	20 (24.1%)	<0.001^2^
Age of diagnosis	1767	56 [48; 63]	83	53 [40;62]	0.042^1^
PTH, pg/ml	1746	168 [114; 284]	77	597 [200; 1422]	<0.001^1^
Calcium total, mmol/l	1637	2.79 [2.67; 2.96]	76	2.95 [2.63; 3.36]	0.003^1^
Albumin-adjusted calcium, mmol/l	217	2.74 [2.63; 2.90]	14	2.81 [2.42; 2.87]	0.869^1^
Calcium ionized, mmol/l	1312	1.38 [1.28; 1.50]	47	1.51 [1.37; 1.68]	<0.001^1^
Phosphorus, mmol/l	1120	0.84 [0.71; 0.98]	49	0.77 [0.67; 0.97]	0.110^1^
Urinary calcium 24 h, mmol/24 h	602	8.48 [5.74; 11.19]	22	6.90 [2.85; 9.45]	0.085^1^
Creatinine, mcmol/l	1112	72 [63; 87]	46	87 [69; 151]	<0.001^1^
GFR, ml/min/1.73m2	1122	81 [65; 96]	47	65 [36; 91]	<0.001^1^
ALP, units/l	851	148.0 [96.3; 272.0]	34	404.0 [130.0; 684.2]	<0.001^1^
Bone complications	1767	845 (47.8%)	83	47 (56.6%)	0.144^2^
Kidney complications	1767	617 (34.9%)	83	47 (56.6%)	<0.001^2^
Cardiovascular diseases during life	1767	743 (42.0%)	83	31 (37.3%)	0.427^2^

^1^U-test; ^2^ Fisher exact test; Bonferroni correction Р0 = 0.05/14 = 0.004. Data are presented by medians and interquartile ranges (Median, IQR [25;75]%). N - Number of people examined. PTH, parathyroid hormone; GFR, estimated glomerular filtration rate; ALP, alkaline phosphatase.

Drug therapy was prescribed in 54% (2665) of cases, where the data was available (n=4934). Among them 57.8% were taking cholecalciferol (34.9% as the monotherapy), 28.7% - bisphosphonates in various regimes (predominantly alendronate 70 mg once a week), 12.7% - cinacalcet, 10.7% - denosumab. The most commonly used combination was cholecalciferol and bisphosphonates (in 12.4% of cases).

## Discussion

The average age of patients in the Registry at the time of diagnosis was 59 years in women and 52 years in men, which is consistent with other studies. There was a significant prevalence among women. This could be explained by the active screening for osteoporosis among postmenopausal women, as well as the lower level of medical care demand among men. The active phase of the disease at the time of data presentation was registered in 82.2% of patients, while in the majority (74.3%) PHPT-associated complications were verified. The predominant clinical manifestation was bone pathology (62.5%), and more often, it was observed in combination with visceral complications (45.7%). There was a fairly high frequency of nephrolithiasis in 44.8% of patients, while nephrocalcinosis was recorded only in 2.8%. This is probably due to the rare use of CT for its verification. Compared to the asymptomatic PHPT, the symptomatic subjects had significantly higher levels of iPTH, serum calcium and daily calciuria, as well as lower phosphorus and GFR levels, so they can be considered as some indicators of the severity of the disease at the time of the initial diagnosis.

In the USA, Canada, and Europe asymptomatic PHPT has been the predominant clinical phenotype over the past 40 years. The prevalence of clinically overt nephrolithiasis has decreased from approximately 80% of patients in earlier series to 7–20% presently. However, in systematically screened patients the diagnostic prevalence may be higher (25–55%) (12). Severe bone manifestations including osteitis fibrosa cystica and skeletal deformities are also uncommon in developed countries. In the USA, <2% of patients have osteitis fibrosa cystica (3).

The situation in the Middle East, Asia and South America is different mainly because the testing for serum calcium is not routine. In Latin America, a 2015 report indicated that more than 50% of patients presented with osteitis fibrosa cystica or nephrolithiasis; other reports confirmed high rates of nephrolithiasis (>40%) (6). Until recently in China there was also a high frequency of manifest forms presented with classical symptoms such as nephrolithiasis and bone pain (7). However, there has been a trend towards more frequent detection of patients with a mild course from about 20 up to 38% of total PHPT cases until 2010 with constant increase in asymptomatic cases after 2007 (7).

A significant predominance of symptomatic forms, same as our data, was noted for the Indian population. Similar to the Russian Registry the Indian Registry has online access from different centers (http://www.indianphptregistry.com), although it is limited to five tertiary centers covering four different geographical regions. The majority of included patients had a manifest form (total 95%) and were characterized by high levels of calcium and PTH. The clinical manifestations dominated by nonspecific symptoms - weakness and fatigue (58.7%), bone disease (56%) and renal stones (31%); pancreatitis and gallstone disease occurred in 12.3% and 11% respectively. The authors concluded that compared to Western countries, Indian patients with PHPT were younger (the mean age was 41 ± 14 years) and biochemical abnormalities showed more significant changes (13).

Epidemiological studies in the Russian Federation are still limited, although interest in PHPT is growing. In 2020, our colleagues from St. Petersburg published the retrospective data on 394 hospitalized patients with PHPT who underwent PTx. As a result, symptomatic PHPT was the dominant form in 56.4% of cases, however asymptomatic disease occurred in 43.6%, which is higher compared to the data of the Russian Registry. This is probably due to the sampling bias because of the better diagnostic and treatment capabilities of the Center compared to the smaller clinics. Skeletal involvement, similar to our results, was more common for women, while the frequency of kidney pathology did not differ in both genders. According to our data, renal disease was more often recorded in men. In contrast to our results, symptomatic and asymptomatic patients did not differ in age. The same as in our study, serum iPTH level was higher in symptomatic patients (P = 0.022). Serum 25(OH)D levels were estimated in some patients and negatively correlated with iPTH (r = -0.294, P = 0.005), ionized Ca (r = -0.268, P = 0.010) and total Ca (r = -0.284, P = 0.014) levels (14).

NPHPT is a condition that can present with intermittent hypercalcemia or may evolve into hypercalcemic PHPT. The prevalence of nPHPT in the literature varies considerably, and it is reported to be between 0.1% and 8.9%. The reason for this variation is the usage of different definitions and methodologies (15). Clinical features of nPHPT can be similar to the ones described in PHPT (skeletal, renal complications, and nonclassical manifestations) (11). NPHPT may also increase cardiovascular risk, probably due to the physiologic effects of PTH and/or serum calcium on the cardiomyocyte, cardiac conduction system and endothelial cells. In particular, nPHPT patients had higher levels of blood pressure than the subjects with normal PTH (16). According to our results, the incidence of nPHPT was 5.8%, which is consistent with other studies. Normocalcemic group did not differ from the hypercalcemic one in the incidence of skeletal and renal disorders, which is probably because the complications of the disease were the reason for the calcium screening. Moreover, the nPHPT group also had a comparable high CVD rate of 43.6%.

The cardiovascular involvement as PHPT complication remains controversial, however a number of data have been published regarding the associations between symptomatic and asymptomatic PHPT with AH, arrhythmias, endothelial dysfunction, glucose metabolism impairment and metabolic syndrome (16). According to our results, CVD in summary was recorded in 48% of patients, while the expected total frequency has been increased with age. The cardiovascular disorders were more typical for the symptomatic PHPT among them AH was detected most often - up to 48.5% in this group. Previously we have demonstrated, that the frequency of AH can vary from 29.7% in people under 50 years of age to 94% in patients over 65 years of age (17). In other studies, this rate is about 40-60%. The high prevalence of AH among patients with PHPT may be due to both the direct effect of hypercalcemia and the direct effects of PTH on cardiomyocytes, however, this requires further study in basic research (18, 19).

Yanevskaya L. et al. also showed the high percentage of CVD in PHPT but unlike our data it was equal between symptomatic and asymptomatic patients (70.7 and 63.4%, P = 0.076). Both age and body mass index were higher in patients with CVD, whether or not they were symptomatic (P < 0.0001), compared to patients with PHPT without CVD. Among CVD the most common manifestation was also AH (63.9%), while heart failure (10.4%) and rhythm disturbances (9.4%) were observed less often (14). According to the Registry, 19.6% of patients with PHPT had a history of coronary heart disease, depending on age, which also exceeds the general population indicators. As in the case of AH, patients with CAD had the more severe disturbances of phosphorus-calcium metabolism.

Hereditary causes account for up to 10% of PHPT cases and include several multiple tumor syndromes (MEN; predominantly type 1, less often 2A and 4; hyperparathyroidism-jaw tumor syndrome) or nonsyndromal forms (familial isolated hyperparathyroidism, familial hypocalciuric hypercalcemia, and neonatal severe PHPT) (20). Several studies of patients with MEN1 syndrome and PHPT have been published, including the Spanish registry, patient series from the French and Belgian GENEM study, Italian (Florentine) and Japanese databases (21–25).

All the mentioned studies were multicenter and included a considerable number of patients ranging from 89 in the Spanish registry and up to 506 patients in the Japanese database. The age of PHPT diagnosis in our group was comparable to the Florentine database and the GENEM study, although the age of onset in the Japanese population was higher (46.8 ± 13.1). In our study, the MEN-1 group included more males than the sporadic group (27.1% vs. 7.7%), but female patients either predominated. Other studies showed similar results, except the Spanish Registry (55% males vs. 45% females).

MEN-1 associated PHPT often manifests with mild elevations of serum calcium and iPTH (26), although there is data that bone disease and urolithiasis in MEN1-related PHPT showed an early onset and higher severity compared to sporadic disease (27). In our study there was a trend towards more severe hypercalcemia in MEN-1 patients where in iPTH, phosphorus and creatinine levels did not differ from sporadic disease. There was no statistical difference in frequency of isolated bone complications between sporadic and MEN-1 groups (33.3% vs. 35.1%, p=0,778), and a trend towards less frequent combination of visceral and bone involvement in the MEN-1 group (29.4% vs. 14.0%, p= 0.012).

Our analysis also showed a higher burden of MEN-1 components compared to other databases, which can be the result of expanded examination in specialized centers, where such patients are accumulated, or, on the other hand, it could be a feature of the Russian MEN-1 syndrome population. Further studies are required. Most patients (77.6%) in our cohort underwent primary surgical treatment, 28.9% (n=24) of these patients relapsed. A limitation of our study is the lack of data on the volume of the operation, although this fact is decisive in relation to the recurrence. In the Mariti et al. group, which exhibited a variable extent of surgical treatment, recurrence was reported in 20% of patients (28).

Patients with PHPT who meet any one of the guideline criteria should be advised to undergo PTx, because surgical removal of hyperfunctioning parathyroid tissue remains the only one definitive cure. Failure to cure could reach 2.5–5%, but the surgeon’s experience is predictive for the outcome (29). Successful PTx leads to normalization of calcium-phosphorus metabolism and eventually to higher BMD, reduced fracture risk and risk of kidney stones. Even in nPHPT, PTx has been associated with beneficial outcomes (10). According to our results, surgery has been performed in 53% of patients with remission achievement in 87%. Such a relatively low percentage of surgical treatment can probably be explained by the lack of dynamic data on patients initially entered into the Registry at the time of the disease manifestation. At the same time, this may be due to the possible refusal of surgery in elderly age groups (the group of patients older than 70 years prevailed in the Registry). There is evidence that PHPT is undertreated in the elderly. Wu B. et al. observed a progressive age-related decline in PTx rate that renders patients aged >70 years unlikely to have definitive treatment, irrespective of comorbidity and eligibility for surgery (30). Wherein PTx is safe in most cases in the elderly and is associated with a significant improvement of well-being, moreover the survival rate seems to be similar to younger patients with PHPT (31, 32).

Most patients, according to the histological examination, had sporadic solitary adenoma. Multiple lesions, predominantly hyperplasia, were more often observed in patients with hereditary forms of the disease. Besides, the online access to the Registry has led to a significant increase in the registration of PC in the Russian Federation. Among surgically treated patients with PHPT the frequency of this pathology has reached at the moment about 4%. Patients with PC had higher levels of iPTH, ionized and total calcium, ALP and the lower levels of GFR and phosphorus. Compared to adenomas, kidney pathology was more often recorded in PC, although there were no differences in bone disorders. Moreover, the data from the Registry was previously used for developing of a mathematical model for preoperative diagnostics of PC which can predict adenoma and PC with positive-predictive value of 100% and 81–92%, respectively (33).

Some PHPT patients are unable to undergo PTX (severe comorbidities, refusing or postponing surgery). In such cases, antiresorptive therapy may be used to prevent bone loss, reduce the fracture risk and manage hypercalcemia (34). In the study group, bisphosphonates were common (28.7%), among them alendronate 70 mg once a week was prescribed predominantly. Denosumab has been used less frequently, predominantly in patients with severe bone complications and life-threatened hypercalcemia. Cinacalcet may be considered to reduce calcium and iPTH serum levels, but its effects on BMD are uncertain (35). In our study, 12.7% of patients received cinacalcet to control hypercalcemia. Vitamin D deficiency may increase the likelihood of a more symptomatic presentation of PHPT and is also associated with increased risk of postoperative hypocalcemia and “hungry bone syndrome” (36). Moreover, vitamin D replacement in subjects with PHPT and coexistent vitamin D deficiency reduce serum PTH significantly without causing hypercalcemia and hypercalciuria in patients with mild and moderate hypercalcemia (37, 38). According to our results, more than 50% of patients received cholecalciferol in various doses. However, the data about the initial and dynamic levels of 25(OH)D is not available.

### Limitations

There is a retrospective study with the limitations inherent to the loss of data inserted to the Registry platform. Moreover, there is a multicenter study. The laboratory parameters were determined in different laboratories, as well as instrumental studies, were performed by various specialists on different equipment. The Registry does not allow estimating the number of patients with asymptomatic visceral complications (e.g., asymptomatic nephrolithiasis). There is missing part of the data in some patient records. The additional restriction is the lack of information about 25(OH) vitamin D. The Registry does not record information on the presence of brown tumors, the volume of surgical treatment.

## Conclusion

The rate of PHPT detection in the Russian Federation has increased in recent years with the start of online registration. The majority of patients had clinically significant hypercalcemia and hypercalciuria, as well as confirmed skeletal and/or renal complications, while the incidence of asymptomatic PHPT without organ involvement was 25.7%. This can indicate delayed diagnosis. Patients with symptomatic form were older and had more severe alterations of phosphorus-calcium metabolism. In addition to the high frequency of classical complications, the Registry revealed a high prevalence of CVD, especially of AH. Patients with AH were significantly older, had higher serum PTH levels and lower GFR compared to normotonic individuals. Genetically confirmed hereditary PHPT was noted in 1.9% of cases (mostly within MEN-1 syndrome). Patients with MEN1-associated PHPT had more severe hypercalcemia compared to those with a sporadic pathology and were distinguished with multiple concomitant endocrine lesions. Surgery was performed in 53.4% of patients with remission achievement in most of them, the relapse/persistence rate did not exceed 13%. Following analysis of various PHPT forms and medical interventions will allow us to improve medical care for people with PHPT.

## Data availability statement

The datasets presented in this article are not readily available because the database is copyrighted. Requests to access the datasets should be directed to kovaleva.elena@endocrincentr.ru.

## Ethics statement

The studies involving human participants were reviewed and approved by the Ethics Committee of the Endocrinology Research Centre(Protocol No. 1 dated 17.01.2018). Written informed consent to participate in this study was provided by all participants. Written informed consent to participate in this study was provided by the participants’ legal guardian/next of kin.

## Author contributions

AKE, NM, GM, and ID contributed to conception and design of the study. NM, AKE, ARE, and EVKo organized the database. ARE and AM performed the statistical analysis. AKE wrote the first draft of the manuscript. JK, EB, IM, AG, RS, EA, EVKa, and ED wrote sections of the manuscript. All authors contributed to manuscript revision, read, and approved the submitted version.

## References

[1] Bilezikian JP. Primary hyperparathyroidism. J Clin Endocrinol Metab (2018) 103:3993–4004. doi: 10.1210/jc.2018-01225 30060226 PMC6182311

[2] Yeh MW Ituarte PHG Zhou HC Nishimoto S Amy Liu I-L Harari A. Incidence and prevalence of primary hyperparathyroidism in a racially mixed population. J Clin Endocrinol Metab (2013) 98:1122–9. doi: 10.1210/jc.2012-4022 PMC3590475 23418315

[3] Wermers RA Khosla S Atkinson EJ Achenbach SJ Oberg AL Grant CS. Incidence of primary hyperparathyroidism in Rochester, Minnesota, 1993-2001: an update on the changing epidemiology of the disease. J Bone Miner Res (2005) 21:171–7. doi: 10.1359/JBMR.050910 16355286

[4] Press DM Siperstein AE Berber E Shin JJ Metzger R Jin J. The prevalence of undiagnosed and unrecognized primary hyperparathyroidism: a population-based analysis from the electronic medical record. Surgery (2013) 154:1232–8. doi: 10.1016/j.surg.2013.06.051 24383100

[5] Walker MD Rubin M Silverberg SJ. Nontraditional manifestations of primary hyperparathyroidism. J Clin Densitom (2013) 16:40–7. doi: 10.1016/j.jocd.2012.11.008 PMC3564501 23374740

[6] Walker MD Silverberg SJ. Primary hyperparathyroidism. Nat Rev Endocrinol (2018) 14:115–25. doi: 10.1038/nrendo.2017.104 PMC6037987 28885621

[7] Zhao L Liu J He X-Y Zhao H Sun L Tao B. The changing clinical patterns of primary hyperparathyroidism in Chinese patients: data from 2000 to 2010 in a single clinical center. J Clin Endocrinol Metab (2013) 98:721–8. doi: 10.1210/jc.2012-2914 23365127

[8] Murray TM Rao LG Divieti P Bringhurst FR. Parathyroid hormone secretion and action: evidence for discrete receptors for the carboxyl-terminal region and related biological actions of carboxyl-terminal ligands. Endocr Rev (2005) 26:78–113. doi: 10.1210/er.2003-0024 15689574

[9] Zavatta G Clarke BL. Normocalcemic primary hyperparathyroidism: need for a standardized clinical approach. Endocrinol Metab (2021) 36:525–35. doi: 10.3803/EnM.2021.1061 PMC8258342 34107603

[10] Bollerslev J Rejnmark L Zahn A Heck A Appelman-Dijkstra NM Cardoso L. European Expert consensus on practical management of specific aspects of parathyroid disorders in adults and in pregnancy: recommendations of the ESE educational program of parathyroid disorders (PARAT 2021). Eur J Endocrinol (2022) 186:R33–63. doi: 10.1530/EJE-21-1044 PMC8789028 34863037

[11] Bilezikian JP Khan AA Silverberg SJ Fuleihan GE Marcocci C Minisola S. Evaluation and management of primary hyperparathyroidism: summary statement and guidelines from the fifth international workshop. J Bone Miner Res (2022) 37:2293–314. doi: 10.1002/jbmr.4677 36245251

[12] Khan AA Hanley DA Rizzoli R Bollerslev J Young JE Rejnmark L. Primary hyperparathyroidism: review and recommendations on evaluation, diagnosis, and management. a Canadian and international consensus. Osteoporos Int (2017) 28:1–19. doi: 10.1007/s00198-016-3716-2 PMC5206263 27613721

[13] Bhadada SK Arya AK Mukhopadhyay S Khadgawat R Sukumar S Lodha S. Primary hyperparathyroidism: insights from the Indian PHPT registry. J Bone Miner Metab (2018) 36:238–45. doi: 10.1007/s00774-017-0833-8 28364324

[14] Yanevskaya LG Karonova T Sleptsov IV Boriskova ME Bakhtiyarova AR Chernikov RA. Clinical phenotypes of primary hyperparathyroidism in hospitalized patients who underwent parathyroidectomy. Endocr Connect (2021) 10:248–55. doi: 10.1530/EC-20-0515 PMC7983481 33416513

[15] Schini M Jacques RM Oakes E Peel NFA Walsh JS Eastell R. Normocalcemic hyperparathyroidism: study of its prevalence and natural history. J Clin Endocrinol Metab (2020) 105:e1171–86. doi: 10.1210/clinem/dgaa084 PMC7069345 32072184

[16] Pepe J Cipriani C Sonato C Raimo O Biamonte F Minisola S. Cardiovascular manifestations of primary hyperparathyroidism: a narrative review. Eur J Endocrinol (2017) 177:R297–308. doi: 10.1530/EJE-17-0485 28864535

[17] Gorbacheva AM Bibik EE Dobreva EA Elfimova AR Pushkareva AS Eremkina AK. Structure of metabolic disorders and cardiovascular disease in patients with primary hyperparathyroidism: a single-center retrospective observational study. Profil meditsina (2022) 25:54. doi: 10.17116/profmed20222508154

[18] Wetzel J Pilz S Grübler MR Fahrleitner-Pammer A Dimai HP von Lewinski D. Plasma parathyroid hormone and cardiovascular disease in treatment-naive patients with primary hyperparathyroidism: the EPATH trial. J Clin Hypertens (2017) 19:1173–80. doi: 10.1111/jch.13064 PMC8030827 28834128

[19] Demirtas D Sumbul HE Demirtas AO Icen YK Gulumsek E Koca H. Morning blood pressure surge increases in patients with hypertensive primary hyperparathyroidism and is independently associated with serum calcium level. Clin Exp Hypertens (2020) 42:86–92. doi: 10.1080/10641963.2019.1590388 30895812

[20] Cetani F Saponaro F Borsari S Marcocci C. Familial and hereditary forms of primary hyperparathyroidism. Front Horm Res (2019) 51: 40–51. doi: 10.1159/000491037. 30641519

[21] Lamas C Navarro E Casterás A Portillo P Alcázar V Calatayud M. MEN1-associated primary hyperparathyroidism in the Spanish registry: clinical characterictics and surgical outcomes. Endocr Connect (2019) 8:1416–24. doi: 10.1530/EC-19-0321 PMC6826168 31557724

[22] Goudet P Cougard P Vergès B Murat A Carnaille B Calender A. Hyperparathyroidism in multiple endocrine neoplasia type I: surgical trends and results of a 256-patient series from groupe d’Etude des néoplasies endocriniennes multiples study group. World J Surg (2001) 25:886–90. doi: 10.1007/s00268-001-0046-z 11572029

[23] Marini F Giusti F Brandi ML. Multiple endocrine neoplasia type 1: extensive analysis of a large database of Florentine patients. Orphanet J Rare Dis (2018) 13:205. doi: 10.1186/s13023-018-0938-8 30428914 PMC6237029

[24] Giusti F Cianferotti L Boaretto F Cetani F Cioppi F Colao A. Multiple endocrine neoplasia syndrome type 1: institution, management, and data analysis of a nationwide multicenter patient database. Endocrine (2017) 58:349–59. doi: 10.1007/s12020-017-1234-4 28132167

[25] Sakurai A Suzuki S Kosugi S Okamoto T Uchino S Miya A. Multiple endocrine neoplasia type 1 in Japan: establishment and analysis of a multicentre database. Clin Endocrinol (Oxf) (2012) 76:533–9. doi: 10.1111/j.1365-2265.2011.04227.x 21950691

[26] Giusti F Tonelli F Brandi ML. Primary hyperparathyroidism in multiple endocrine neoplasia type 1: when to perform surgery? Clinics (2012) 67:141–4. doi: 10.6061/clinics/2012(Sup01)23 PMC3328829 22584719

[27] Mele C Mencarelli M Caputo M Mai S Pagano L Aimaretti G. Phenotypes associated with MEN1 syndrome: a focus on genotype-phenotype correlations. Front Endocrinol (Lausanne) (2020) 11:591501. doi: 10.3389/fendo.2020.591501 33312161 PMC7708377

[28] Marini F Giusti F Cioppi F Maraghelli D Cavalli T Tonelli F. Bone and mineral metabolism phenotypes in MEN1-related and sporadic primary hyperparathyroidism, before and after parathyroidectomy. Cells (2021) 10:1895. doi: 10.3390/cells10081895 34440663 PMC8391385

[29] Stavrakis AI Ituarte PHG Ko CY Yeh MW. Surgeon volume as a predictor of outcomes in inpatient and outpatient endocrine surgery. Surgery (2007) 142:887–99. doi: 10.1016/j.surg.2007.09.003 18063073

[30] Wu B Haigh PI Hwang R Ituarte PHG Liu I-LA Hahn TJ. Underutilization of parathyroidectomy in elderly patients with primary hyperparathyroidism. J Clin Endocrinol Metab (2010) 95:4324–30. doi: 10.1210/jc.2009-2819 PMC2936062 20610600

[31] Stechman MJ Weisters M Gleeson FV Sadler GP Mihai R. Parathyroidectomy is safe and improves symptoms in elderly patients with primary hyperparathyroidism (PHPT). Clin Endocrinol (Oxf) (2009) 71:787–91. doi: 10.1111/j.1365-2265.2009.03540.x 19222492

[32] Norenstedt S Ekbom A Brandt L Zedenius J Nilsson I-L. Postoperative mortality in parathyroid surgery in Sweden during five decades: improved outcome despite older patients. Eur J Endocrinol (2009) 160:295–9. doi: 10.1530/EJE-08-0523 19042978

[33] Krupinova JA Elfimova AR Rebrova OY Voronkova IA Eremkina AK Kovaleva EV. Mathematical model for preoperative differential diagnosis for the parathyroid neoplasms. J Pathol Inform (2022) 13:100134. doi: 10.1016/j.jpi.2022.100134 36268079 PMC9577121

[34] Ye Z Silverberg SJ Sreekanta A Tong K Wang Y Chang Y. The efficacy and safety of medical and surgical therapy in patients with primary hyperparathyroidism: a systematic review and meta-analysis of randomized controlled trials. J Bone Miner Res (2022) 37:2351–72. doi: 10.1002/jbmr.4685 36053960

[35] Dillon ML Frazee LA. Cinacalcet for the treatment of primary hyperparathyroidism. Am J Ther (2011) 18:313–22. doi: 10.1097/MJT.0b013e3181bdc3d0 20228675

[36] Walker MD Cong E Lee JA Kepley A Zhang C McMahon DJ. Vitamin d in primary hyperparathyroidism: effects on clinical, biochemical, and densitometric presentation. J Clin Endocrinol Metab (2015) 100:3443–51. doi: 10.1210/jc.2015-2022 PMC4570160 26079779

[37] Acharya R Kopczynska M Goodmaker C Mukherjee A Doran H. Vitamin d repletion in primary hyperparathyroid patients undergoing parathyroidectomy leads to reduced symptomatic hypocalcaemia and reduced length of stay: a retrospective cohort study. Ann R Coll Surg Engl (2022) 104:41–7. doi: 10.1308/rcsann.2021.0078 PMC10335014 34727512

[38] Shah VN Shah CS Bhadada SK Rao DS. Effect of 25 (OH) d replacements in patients with primary hyperparathyroidism (PHPT) and coexistent vitamin d deficiency on serum 25(OH) d, calcium and PTH levels: a meta-analysis and review of literature. Clin Endocrinol (Oxf) (2014) 80:797–803. doi: 10.1111/cen.12398 24382124

